# Genetic structure of Mexican lionfish populations in the southwest Gulf of Mexico and the Caribbean Sea

**DOI:** 10.1371/journal.pone.0222997

**Published:** 2019-10-01

**Authors:** Elizabeth Labastida-Estrada, Salima Machkour-M’Rabet, Laura Carrillo, Yann Hénaut, Delma Nataly Castelblanco-Martínez

**Affiliations:** 1 Laboratorio de Ecología Molecular y Conservación, Departamento de Conservación de la Biodiversidad, El Colegio de la Frontera Sur, Chetumal, Quintana Roo, Mexico; 2 Departamento de Sistemática y Ecología Acuática, El Colegio de la Frontera Sur, Chetumal, Quintana Roo, Mexico; 3 Laboratorio de Conducta Animal, El Colegio de la Frontera Sur, Chetumal, Quintana Roo, Mexico; 4 Consejo Nacional de Ciencia y Tecnología / Universidad de Quintana Roo, Chetumal, Quintana Roo, Mexico; Leibniz-Institute of Freshwater Ecology and Inland Fisheries, GERMANY

## Abstract

The recent expansion of the invasive lionfish throughout the Western Hemisphere is one of the most extensively studied aquatic invasions. Molecular studies have improved our understanding of larval dispersal, connectivity, and biogeographical barriers among lionfish populations, but none have included Mexican localities, an important area for the larval dispersal of *Pterois volitans* through the Western Caribbean and the Gulf of Mexico. Here, we present a genetic analysis of lionfishes collected along Mexican coasts, examining their connectivity with other Caribbean localities (Belize, Cuba, Puerto Rico) and the role of ocean currents on population structure. We collected 213 lionfish samples from seven locations comprising four countries. To evaluate genetic structure, mitochondrial control region and nuclear inter-simple sequence repeat markers were used. We found that lionfish collected along Mexican coasts show a similar haplotype composition (H02 followed by H01 and H04) to other Caribbean locations, and the H03 rare haplotype was not found. Haplotype composition in the southwest Gulf of Mexico suggests a discontinuity between the southern and northern areas of the Gulf of Mexico. The southern area clustered more strongly to the Caribbean region, and this is supported by the complexity of water circulation in the semi-enclosed region of the Gulf of Mexico. Mitochondrial genetic diversity parameters show small values, whereas nuclear markers produce medium to high values. Only nuclear markers highlighted significant genetic differentiation between the southwest Gulf of Mexico and Caribbean region, confirming a phylogeographic break between both regions. Separate analysis of Caribbean locations indicates restricted larval exchange between southern and northern regions of the Mesoamerican Barrier Reef System, potentially in response to regional oceanographic circulation.

## Introduction

Biological invasion is significant threat to biodiversity and it is a result of humans [1] permeating biogeographic barriers [2] and thus favoring species dispersal [3–4]. Aquatic environments are particularly affected by invasive species [2], as highlighted by Roman and Darling [5] who list thousands of aquatic (estuarine, freshwater, and marine) invasive species that have dispersed throughout the world. The red lionfish (*Pterois volitans* L. 1758; Scorpaeniformes, Scorpaenidae) is an important and well-documented case of an aquatic invasive species. This species has recently invaded the Western Atlantic, and has rapidly dispersed throughout the Caribbean and Gulf of Mexico (see [6] for a review of larval dispersal history). Due to its quick and successful dispersal rate, and its impact on biodiversity and dynamic of coral reef ecosystems [7], a plethora of lionfish studies have emerged. Topics include ecology, behaviour, parasitology, management, genetic, among others [6, 8–14].

**Fig 1:**
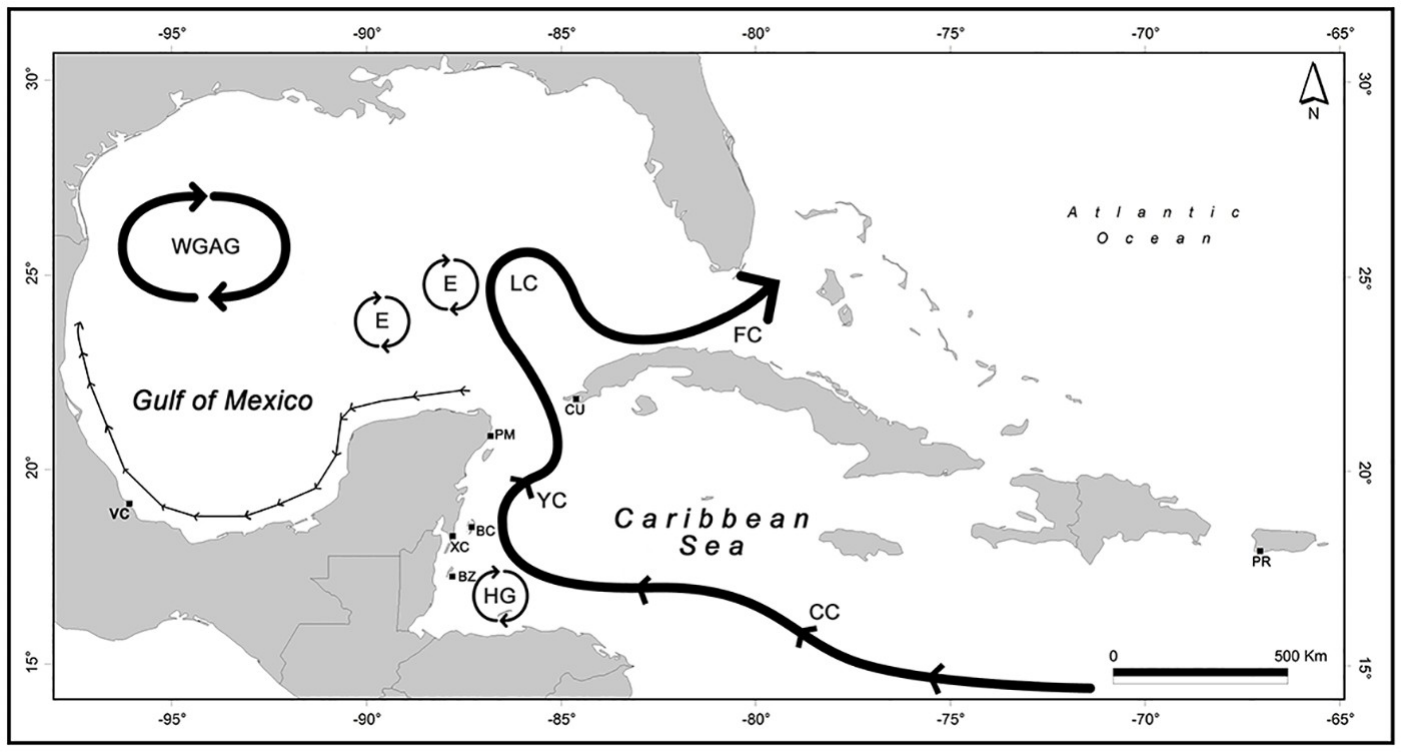
Map of the sample locations for this study in the Caribbean Sea and the Gulf of Mexico, including different oceanographic features. Banco Chinchorro (BC), Puerto Morelos (PM), Veracruz (VC), Xcalak (XC), Cuba (CU), Belize (BZ), Puerto Rico (PR), Hondura Gyre (HG), Caribbean Current (CC), Yucatan Current (YC), Loop Current (LC), Florida Current (FC), Anticyclonic and Cyclonic eddies (E), Campeche Bank Gyre or Campeche Bay gyre (CB), Western Gulf Anticyclonic Gyre (WGAG), and small thin arrow indicates circulation over the Southern Gulf shelf.

Numerous molecular techniques have been developed to study lionfish taxonomy, ecology, biogeography, and population genetics [6, 15–20]. Genetics studies have improved our understanding about patterns of gene flow and larval dispersal, relationships among newly established populations, and identifying phylogeographic barriers ([21] for a review). Studies based on the mitochondrial DNA (mtDNA) control region have identified only nine haplotypes (H01-H09) within the invaded range whereas a total of 36 haplotypes (H10-H45) have been determined for the native range in Western Indonesia and the Philippines, reflecting a strong founder effect [8]. The Caribbean region presents the most important reduction of genetic diversity with just four haplotypes (H01-H04) [8–9] of which three (H01, H02 and H04) have been recently identified in the northern Gulf of Mexico (Texas, Mississippi, and Florida) [21]. Additionally, significant genetic structure among the three principal regions (northern Gulf of Mexico *versus* Caribbean Sea *versus* north Atlantic) has been demonstrated; however, the species has not exhibited genetic structure among the Gulf of Mexico and Caribbean region [21]. A similarity in haplotypes between the Caribbean and the Gulf of Mexico and a substantial genetic dissimilarity between the latter and North Atlantic has led previous authors to conclude that there is no detectable gene flow from the north Atlantic to the Gulf of Mexico and therefore red lionfish populations in the Gulf of Mexico originated from the Caribbean region. They suggest that the swiftly flowing Florida Current significantly limits opportunities for dispersal (larvae form [22]) from the Atlantic into the Gulf of Mexico. Lastly, the same authors suggest that there is a reduction of gene flow between the Gulf of Mexico and the Caribbean region populations (dashed line on Fig 1 [21]). This genetic break between these marine regions was also demonstrated for other species as for blacktip sharks (*Carcharhinus limbatus*, Müller & Henle 1839) [23], and the queen conch (*Strombus gigas* L.1758) [24]. Although studies have shown or suggested genetic break for marine species between the Gulf of Mexico and the Caribbean region, it is important to consider the location of samples inside the Gulf because recent researches [25–26] suggest low connectivity between the northern and the southern regions of the Gulf. This could have important genetic implication for the lionfish connectivity between Caribbean and the different areas of the Gulf of Mexico. A recent study based on nuclear genome markers (single nucleotide polymorphisms; SNPs) indicates genetic homogeneity among the northern Gulf of Mexico (Texas, Louisiana, Alabama, western coast of Florida), the Florida Keys, and the eastern coast of Florida [6].

Molecular information on lionfish from the Caribbean region is extensive; however, there is no research on specimens from the Mexican coast, significantly along the coast of the Yucatan Peninsula that encompasses a large part of the Mesoamerican Reef and protected areas such as Puerto Morelos Reef National Park, Biosphere Reserve of Banco Chinchorro, Xcalak Reef National Park, among others. This region is very important for gaining an insight into the dispersal patterns of *P*. *volitans* larvae through the Caribbean Sea to the Gulf of Mexico. Some authors have suggested that the key dispersion factor for lionfish larvae are marine currents [27–28], and there is a growing body of evidence that circulation patterns determine connectivity among marine populations [29–30]; consequently, larvae originating from the eastern Caribbean travel along the Cayman Current towards the Quintana Roo coast, close to the latitude of Banco Chinchorro (southern zone of the Mexican Caribbean). This current subsequently becomes the Yucatan Current, flowing northwards while hugging the eastern coast of the Yucatan Peninsula, then developing into the Loop Current that flows into the Gulf of Mexico. The Loop Current finally exits the Gulf of Mexico as the Florida Current and out into the Atlantic Ocean (Fig 1 in [31]). The marine current patterns in the Gulf of Mexico are dominated by the Loop Current and eddy shedding which flow in a westerly direction [32]. The Yucatan Current is well known as one of the strongest currents in the world and represents an important flow of water from the Caribbean Sea to the Gulf of Mexico [33–34]. Recent research has described circulation along the Mesoamerican Reef System as having three different regimes: a northern region with a well-defined Yucatan Current, a southern region with low and variable velocity field with even counter-currents, and a mesoscale cyclonic gyre known as the Honduras gyre (Fig 3 in [31]).

For the first time, we present genetic data from the Mexican coast (Caribbean Sea and the southwest Gulf of Mexico) using mtDNA control region and inter-simple sequence repeat (ISSR) marker, a high polymorphic and contemporary nuclear marker (see review for characteristics of ISSR in [24]). The mtDNA control region has been selected given that it has been the predominant genetic marker used in phylogeographic and genetic structure studies of lionfish, thus allowing a direct comparison of results; it is recommended for research on phylogeographic patterns in marine species and historical processes related to genetic diversity distribution [35]. Nevertheless, the identification of fine-scale connectivity for lionfish invasion (e.g., between the Mexican Caribbean and the Mexican coast of the Gulf of Mexico) using only one mtDNA marker could be jeopardized due to the lower variation of mtDNA, and therefore the use of nuclear loci is recommended [6, 8]. In view of this, we used a nuclear dominant marker on the same dataset to describe the regional genetic structure that seemed to be absent following the mtDNA results [21]. Nuclear ISSR markers are particularly efficient for populations genetic studies of marine organisms, even at small spatial scales [24, 35]. A recent study based on nuclear genome markers (SNPs) indicates the possibility of a rapid genetic change over space and time for lionfish invasion [6]; therefore, to avoid the probable effect of time on the genetic characteristic of lionfish in the invaded region, all samples were collected over a short time period and previous results of mtDNA were not incorporated in our analysis.

Here, we present the first genetic study of red lionfish collected along the Mexican coast. We focus on the following questions: (1) Do Mexican populations (Caribbean Sea and southwest Gulf of Mexico) present the same mitochondrial haplotypes as other Caribbean populations, and do they present any signs of the founder effect?, (2) Do ISSR nuclear markers will successfully demonstrate the possible phylogeographic break between the Mexican Caribbean and the southwest Gulf of Mexico, where the mitochondrial control region failed?, and (3) Does the genetic connectivity among the Caribbean Sea and the Gulf of Mexico populations of lionfish reflect the oceanic current dynamic?

## Materials and methods

### Ethics statement

Lionfish samples of Mexico were obtained from fishermen with the authorization from the relevant Natural Protected Areas (see Acknowledgments for details). No permits were required to collect specimens in Puerto Rico, and all samples were obtained from fishermen. Permission to collect and export samples from Cuba was provided by the “Instituto de Medicina Veterinaria de Cuba”. In Belize, collection of samples was conducted with a Marine Scientific Research Permit 000030–14, granted by the Belize Fisheries Department, Ministry of Forestry, Fisheries and Sustainable Development. Experimental protocol was approved by Ethical Committee from the “El Colegio de la Frontera Sur”, Mexico.

### Lionfish samples and DNA extraction

Red lionfish (n = 213) were collected from a total of seven sample sites in four countries. Four sample sites were situated on the Mesoamerican Reef on the Mexican Coast while the remaining three were in Belize, Cuba, and Puerto Rico (see Fig 1 and Table 1 for details on sites and collection methods). Muscle tissues were stored in 96% ethanol, or dimethyl sulfoxide (DMSO) tissue buffer following their origin, at 4°C. DNA extraction in addition to quality and quantity were processed following Labastida et al. [17]. DNA concentration was standardized at 20 ng μL^-1^ to optimized PCR efficiency across all samples.

**Table 1:** Information of red lionfish samples collected at seven localities in the Caribbean Sea and the Gulf of Mexico.

Country	Locality	N	GC	Collect method
**Mexico (MX)**	Banco Chinchorro Biosphere Reserve (BC)^a^	40	18°34’N, 87°18’W	Scuba diver
	Puerto Morelos Reef National Park (PM)^a^	34	20°54’N, 86°49’W	Fishermen
	Xcalak National Reef Park (XC)^a^	35	18°20’N, 87°47’W	Scuba diver
	Veracruz Coral Reef System National Park (VE)^b^	21	19°10’ N, 96°05’W	Scuba diver
**Cuba (CU)**	Guanahacabibes Biosphere Reserve (CU)^d^	16	21°51’N, 84°37’W	Scuba diver
**Belize (BZ)**	Turneffe Atoll Marine Reserve (BZ)^c^	38	17°18'N, 87°48'W	Fishermen
**Puerto Rico (PR)**	La Parguera (TR)^e^	29	17°57’N, 67°03’W	Snorkeling and scuba diver

Number of individuals collected (N), geographic coordinates (GC). Superscript letters indicate collectors and their affiliation: E. Labastida-Estrada (a), Tomás Camarena Luhrs for Veracruz Coral Reef System National Park (b), Nataly Castelblanco Martínez for Oceanic Society-Belize (c), Dorka Cobián for ECOVIDA-Cuba, Cuba National Aquarium (d), and Ximena Vélez-Zuazo for Puerto Rico University (e).

### PCR amplification and genotyping

The fragment of 679 pb of mtDNA control region was obtained using the primer LionA-H (5’-CCATCT TAA CAT CTT CAG TG-3’) and LionB-H (5’-CAT ATC AAT ATG ATC TCA GTAC-3’) [36]. PCR reactions were performed in a final volume of 17μl on a Bio-Rad T100 Thermal Cycler with PCR cocktail and cycle conditions as described by Toledo-Hernández et al. [36]. The concentration of PCR products was evaluated using electrophoresis in 2% agarose gel in 1X TAE buffer and a 100 bp DNA ladder (Promega) as reference. Bands were detected using GelRed (Biotum), post-staining method (20 μl of GelRed + 500 μl Blue/Orange Loading Dye, Promega), under UV light, and digitalized with an Imaging System (Photodoc-IT65, UVP). Amplified fragments were sent for purification and sequencing to Macrogen (Seoul, Korea). GenBank accession numbers for mtDNA control region sequences are provided in S1 Table.

PCR amplification and electrophoresis conditions of the anchored at 3’ end nuclear gene (ISSR) were conducted for 213 individuals, following Labastida et al. [17] using the same five primers: (GACA)4WB, (CA)8AC, (CA)8AG, (AG)8YC, (GAG)5GC. To ensure the genotyping accuracy obtained during our experiments, we used negative control and replicates (5% of the full dataset) as suggested for dominant genetic markers [37–38] which allow us to determine the genotyping error rate [38–39]. The mean error rate per locus was 2.35% [(CA)_8_AC: 1.83%; (GACA)_4_WB: 5.16%; (GAG)_5_GC: 0.75%; (AG)_8_YC: 3.2%; (CA)_8_AC: 0.82%] what goes into the average reported in others studies (range 0.2%-15% see [39]) indicating that our data were suitable for analysis.

### Data analysis of mtDNA control region

The forward and reverse sequences were assembled and edited using Sequencher 4.1 (Gene Codes, Ann Arbor, MI USA), and sequences were subsequently aligned with Muscle [40] in Mega 6.0 [41].

To identify haplotypes in the studied region, our dataset was compiled and compared with the nine haplotypes reported for *Pterois volitans* in the invaded area [15] using DnaSP 5 [42]. Genetic diversity at each locality was estimated by computing haplotype diversity (*h*, also called genetic diversity) and nucleotide diversity (π) with Arlequin 3.5 [43].

Genetic differentiation among populations was evaluated by applying the haplotype population pairwise *Φ*_*ST*_ values based on Tamura and Nei distances (10,000 permutations) using Arlequin 3.5, and the sequential Bonferroni correction, as implemented by Rice [44], was used to correct the multiples tests of significance. Additionally, we processed a non-parametric analysis of molecular variance (AMOVA) (10,000 permutations) following the Tamura and Nei (TrN) model, the best-fit model for nucleotide substitution as selected by the corrected Akaike Information Criterion (AIC) in JModelTest 2.1.9 [45–46] and available in Arlequin 3.5 (10,000 permutations). To identify the most plausible phylogeographic breaks in the study region, different AMOVA scenarios were tested to maximize the percentage of variance among regions based on previous knowledge and hypothesis of phylogeographic breaks for lionfish (see Table 2 for a synthesis of all scenarios): a) the first scenario considers high connectivity among all localities, therefore there is no phylogeographic break between the southwest Gulf of Mexico and the Caribbean region, b) the second scenario considers the presence of a barrier that decreases connectivity between the southwest Gulf of Mexico and the Caribbean region, c) the third scenario considers two phylogeographic breaks, the first isolating the southwest Gulf of Mexico and the second separating coastal localities of the Mesoamerican reef (including Xcalak, Puerto Morelos and Belize) from other Caribbean localities (Banco Chinchorro, Cuba, and Puerto Rico), and finally d) the fourth scenario considers two phylogeographic breaks, the first separating the southwest Gulf of Mexico and the second separating the Mexican and Belizean localities from the remainder (Cuba and Puerto Rico). Finally, to identify the phylogenetic relationship among haplotypes, a spanning network was constructed under the parsimony criterion in TCS 1.21 [47].

**Table 2:** Scenarios proposed for AMOVA with mtDNA and ISSR genetic markers.

Phylogeographic break	Scenario	N groups	Localities considered
**No break** All localities grouped	1	1	BC-PM-VE-XC-CU-BZ-PR
**One break** Break: between southwest GM *vs* Caribbean region	2	2	GR1: VE GR2: BC-PM-XC-CU-BZ-PR
**Two breaks** Break 1: between southwest GM *vs* Caribbean region Break 2: between coastal localities of Caribbean region *vs* the rest of Caribbean	3	3	GR1: VE GR2: XC-PM-BZ GR3: BC-CU-PR
**Two breaks** Break 1: between southwest GM *vs* Caribbean region Break 2: between Mexican and Belizean localities of the Caribbean *vs* the rest of Caribbean region considered in this study	4	3	GR1: VE GR2: BC-XC-PM-BZ GR 3: CU-PR

Number of groups (N groups), for localities abbreviation see Table 1.

### Data analysis of ISSR nuclear gene

Scoring of ISSR loci was made manually considering 1 for presence and 0 for absence of bands. This information allows the construction of a binary matrix over all samples and all primers. Only bands which could be scored consistently, with clear and unambiguous patterns among all localities, as well as individuals presenting genetic information for all primers, were kept for analysis. All ISSR data are available in S2 Table.

Candidate loci under natural selection were assessed using Bayescan 2.1 [48], and the final genetic ISSR database was constructed without loci potentially under natural selection. Genetic diversity was evaluated with percentage of polymorphism (*P*), Nei’s gene diversity (*h*), total genetic diversity in the pooled samples (*H*_*T*_), mean diversity within each locality (*H*_*S*_), proportion of genetic diversity distributed among all localities (*G*_*ST*_) using Popgene 1.31 [49]. To test the difference in genetic diversity among locations, we applied a nonparametric Kruskal-Wallis test (Kolmogorov-Smirnov test resulted in non-normality of data) considering *h* values at each loci as variables. Those statistics were processed by Statistica 7.0.

Differentiation between localities was tested with the population pairwise *Phi*_*PT*_ statistic, an analogue of Wright’s *F*_*ST*_, determined for all pairs of populations with 9999 permutations using GenAlEx 6.4 [50–51], and the level of significance was adjusted using the sequential Boneferroni correction [44]. Following the same methodology applied for mtDNA, we implemented an analysis of molecular variance (AMOVA) (9999 permutations) to identify the more plausible phylogeographic breaks in the study region, maximizing the percentage of variance among regions by applying the same AMOVA scenarios as for mtDNA (Table 2). Furthermore, to determine possible genetic structure within the Caribbean, an AMOVA analysis was carried out for this region only (without the Veracruz sample site) considering different scenarios of break(s); in addition, an IBD (isolation-by-distance) analysis was tested using the Mantel test. Lastly, to provide evidence of a possible break without priori scenarios, we processed a principal coordinate analysis (PCoA). All analyses were realized using GenAlEx 6.4.

## Results

### Mitochondrial control region

High quality mtDNA control region sequences were obtained for a total of 176 lionfish individuals in the studied area, producing a final alignment length of 679 bp. The number of polymorphic sites varies from three to nine, determining a haplotype number of two or three, depending on the locality (Table 3). As previously shown in the Caribbean region, the most common haplotype observed in the sampled localities was H02, representing a total of 72%, followed by H01 (25%) and H04 (3%). Particularly, the Mexican coast sites showed similar values of haplotype frequencies (Table 3), and two localities did not present the H04 haplotype (Banco Chinchorro and Veracruz). The rare Caribbean haplotype (H03) was not found in any of the Mexican samples despite the high number of individuals analyzed (n = 101; Table 3). All haplotype and nucleotide diversity parameters were low (*h*: 0.393–0.480 and *π*: 0.00177–0.00230; Table 3) as expected for an invasive region. The lowest values were observed for Veracruz (southwest Gulf of Mexico) and Cuba localities.

**Table 3:** Genetic diversity indices for mitochondrial control region and nuclear ISSR markers for the red lionfish, *Pterois volitans*, in the Caribbean Sea and the Gulf of Mexico regions.

	mtDNA control region							Nuclear ISSR			
Locality/Country	n_1_	*Hap*	*h*_*1*_	*π*	H01	H02	H04	n_2_	N	% *P*	*h*_*2*_ (±SD)
**BC/MX**	25	2	0.480	0.00212	9	16	0	40	105	77.9	0.246(±0.183)^ab^
**PM/MX**	30	3	0.393	0.00227	5	23	2	34	109	74.3	0.239(±0.195)^ab^
**VE/MX**	19	2	0.409	0.00181	5	14	0	21	90	56.0	0.188(±0.203)^a^
**XC/MX**	27	3	0.453	0.00230	7	19	1	35	104	69.7	0.218(±0.198)^ab^
**TOTAL Mexico**	**101**	**3**	**0.429**	**0.00213**	**26**	**72**	**3**	**130**	**109**	**87.2**	**0.253(±0.174)**
**GUA/CU**	16	2	0.400	0.00177	4	12	0	14	101	70.6	0.246(±0.199)^ab^
**TUR/BZ**	30	3	0.393	0.00227	5	23	2	38	108	86.2	0.271(±0.180)^b^
**PA/PR**	29	2	0.443	0.00196	9	20	0	28	103	70.6	0.224(±0.196)^ab^
**TOTAL ALL**	**176**	**3**	**0.418**	**0.00207**	**44**	**127**	**5**	**210**	**109**	**93.6**	**0.261(±0.170)**

Number of individuals used for mitochondrial control region analysis (n_1_), number of haplotypes (*Hap*), haplotype diversity (*h*_*1*_), nucleotide diversity (π), haplotype frequencies observed (H01, H02 and H04), number of individuals used for nuclear ISSR analysis (n_2_), number of ISSR bands (N), percentage of polymorphism (% *P*), Nei’s genetic diversity (*h*_*2*_), and standard deviation (SD). Letters following means of *h*_*2*_ represent intergroup differences (Kruskal-Wallis test). For localities and country abbreviation see Table 1.

All haplotype population pairwise *Φ*_*ST*_ values were very low and not significant (Table 4). AMOVA scenarios did not detect a break between the southwest Gulf of Mexico and the Caribbean region (Table 5); however, the maximum percentage of variation was obtained under scenario 3 (see Table 2 for details about scenarios), with the pooling of the Banco Chinchorro, Cuba, and Puerto Rico sites, whereas coastal localities (Xcalak, Puerto Morelos, and Belize) were separated from the rest of Caribbean region.

**Table 4:** Population pairwise differentiation (*Φ*_*ST*_ values) based on mtDNA control region sequences (below diagonal; negative values could be considered as 0), and population pairwise differentiation (*Phi*_*PT*_ values) based on nuclear ISSR markers (above diagonal) for the red lionfish in the Caribbean Sea and the Gulf of Mexico.

	**BC**	**PM**	**VE**	**XC**	**CU**	**BZ**	**PR**
**BC**		0.011	0.236**	0.018*	0.022*	0.039**	-0.004
**PM**	0.021		0.250**	0.049**	0.005	0.031**	0.010
**VE**	-0.026	-0.023		0.214**	0.255**	0.237**	0.294**
**XC**	-0.023	-0.022	-0.041		0.071**	0.080**	0.041**
**CU**	-0.024	-0.031	-0.060	-0.044		0.016	0.031*
**BZ**	0.021	-0.034	-0.023	-0.022	-0.031		0.038**
**PR**	-0.032	0.001	-0.039	-0.030	-0.041	0.001	

*P < 0.05 and

** *P* < 0.002 after Bonferroni correction (initial alpha 0.05/21 = 0.002). For localities abbreviations see Table 1.

**Table 5:** Analysis of molecular variance (AMOVA) using mtDNA control region and ISSR nuclear markers, testing different scenarios of proposed phylogeographic breaks for the red lionfish in the Caribbean Sea and the Gulf of Mexico.

	mtDNA region control				Nuclear ISSR			
	df	% variation	*F values*	*p*	df	% variation	*Phi* values	*p*
**Scenario 1**								
Among populations	6	-2.03	*F*_ST_: -0.20	ns	6	8	*Phi*_PT_: 0.084	***
Within populations	169	102.03			203	92		
**Scenario 2**								
Among groups	1	-1.72	*F*_CT_: -0.017	ns	1	20	*Phi*_RT_: 0.205	***
Among populations within groups	5	-1.66	*F*_SC_: -0.016	ns	5	3	*Phi*_PR_: 0.033	***
Within populations	169	103.39	*F*_ST_: -0.033	ns	203	77	*Phi*_PT_: 0.232	***
**Scenario 3**								
Among groups	2	1.33	*F*_CT_: 0.013	ns	2	7	*Phi*_RT_: 0.068	***
Among populations within groups	4	-2.93	*F*_SC_: -0.029	ns	4	3	*Phi*_PR_: 0.038	***
Within populations	169	101.60	*F*_ST_: -0.016	ns	203	90	*Phi*_PT_: 0.103	***
**Scenario 4**								
Among groups	2	-0.75	*F*_CT_: -0.007	ns	2	8	*Phi*_RT_: 0.083	***
Among populations within groups	4	-1.57	*F*_SC_: -0.015	ns	4	4	*Phi*_PR_: 0.039	***
Within populations	169	102.33	*F*_ST_: 0.023	ns	203	88	*Phi*_PT_: 0.118	***

For details about scenarios see Table 2. Level of significance:

*** *P* < 0.001, not significant (ns). Negative values for AMOVA could be assimilated as 0. The highlighted scenarios in grey show the highest percentage of molecular variance. Abbreviations of localities see Table 1.

As suggested by the AMOVA analysis, the haplotype network (S1 Fig) did not permit the detection of population structure since Mexican populations shared the more common haplotypes.

### ISSR nuclear gene

The five ISSR molecular markers resulted in a total of 110 clear and unambiguous bands (loci) for 210 individuals among the seven localities. Analysis for candidate loci under natural selection identified one locus under “very strong” evidence for selection, and the alpha value (α = 1.47) suggests diversifying selection. Consequently, the locus under selection was excluded from our dataset. Private bands (also termed diagnostic/signature bands: bands observed in all individuals of one species, or populations, and not in the others) obtained by ISSR were commonly used to distinguished related species [52–54]; we do not observe private band which could indicate that all individuals belong to the same species.

Considering the whole dataset, genetic diversity parameters were high (*P* = 93.6% and *h* = 0.261±0.170) even for Mexican localities (*P* = 87.2% and *h* = 0.253±0.174). At the locality level, genetic diversity parameters (Table 3) varied considerably with lower values for Veracruz (southwest Gulf of Mexico) (*P* = 56% and *h* = 0.188) and higher values for the Belize locality (*P* = 86% and *h* = 0.271). The Kruskal-Wallis test indicated a significant difference among Nei’s gen diversity (*h*) (*H*_6,763_ = 12.89, *P* = 0.045), and the multiple comparison test detected significant difference between the Veracruz and Belize localities. Total variation (*H*_T_) was 0.263 (±0.028) and mean genetic variation within samples (*H*_S_) was 0.233 (±0.024). Coefficient of genetic differentiation (*G*_ST_) was 0.113.

**Fig 2:**
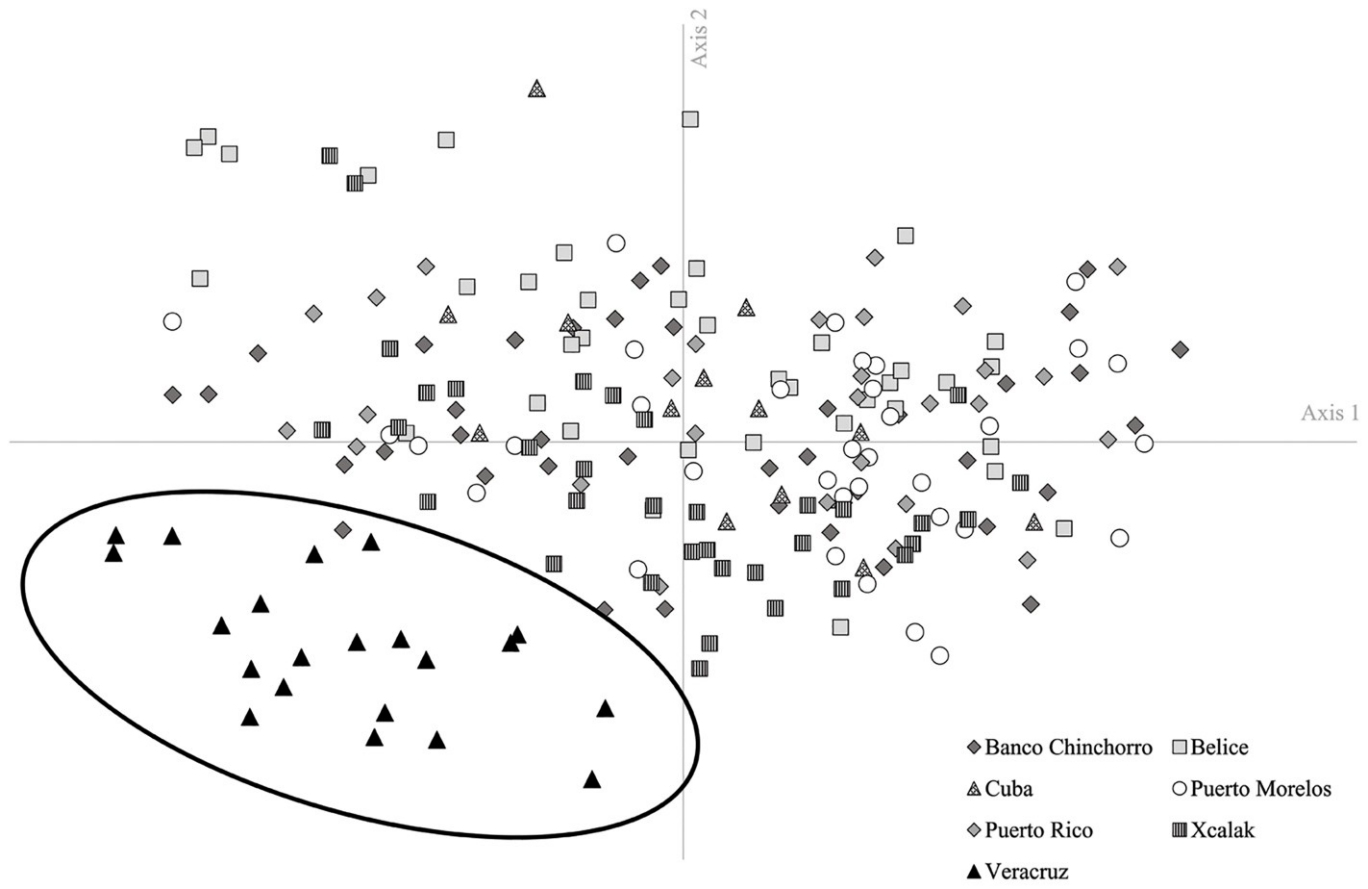
Principal coordinate analysis (PCoA) for *Pterois volitans* based on ISSR nuclear markers differentiating the Gulf of Mexico (encircled locality) from the Caribbean region.

Results for population pairwise differentiation *Phi*_*PT*_ values (Table 4) revealed the highest (> 0.2) and very significant values between the Veracruz locality (southwest Gulf of Mexico) and all other sites, and although other values were significant, they remain very weak comparatively (< 0.08). AMOVA and PCoA analyses confirmed the results obtained by population pairwise differentiation. AMOVA results showed a clear and highly significant difference (20%, *P* < 0.001) between the southwest Gulf of Mexico and the Mexican Caribbean region as indicated by the most probable scenario (number 2) of phylogeographic break (Table 5; details in Table 2 for scenario and S3 Table for complete results). The high percentage of variance among localities (scenario 1; 8%) or among regions (scenario 3 and 4; 7% and 8% respectively) were essentially due to results from the Veracruz locality; therefore, a new dataset (without individuals from Veracruz) was generated in order to provide evidence of possible structure in the Caribbean region. These results (S3 Table) revealed a lower but significant reduction of connectivity between the sample sites at Xcalak (Mexico) and Belize and the remainder of the Caribbean localities considered in this study. The PCoA (Fig 2) revealed a clear separation of the Veracruz locality from the rest of the Caribbean localities. Finally, IBD analysis was significant for the Caribbean region (Mantel test: *r*^2^ = 0.052, *P* = 0.04; S2 Fig), but not significant (Mantel test: *r*^2^ = 0.005, *P* = 0.06; S1 Fig) when the Puerto Rico locality was eliminated.

## Discussion

The haplotype frequencies obtained in this study for the Caribbean region are consistent with previous studies (S4 Table; review in [9]), presenting a dominance of H02 (72%) and H01 (25%) haplotypes and a low frequency for the rare H04 haplotype (3%). These new results suggest that haplotype frequency equilibrium has been reached in this region, thus supporting the idea that the most likely explanation for disparities in haplotype frequencies (principally observed for H03 and H04) is sampling bias due to low sample size rather than temporal variation as suggested by Butterfield et al. [9]. Almost ten years after the first important genetic study on invasive lionfish (based on sample dates from [8]), haplotype frequencies in the Caribbean region have not changed, indicating that there has not been a sufficiently large new invasion wave from the north Atlantic to substantially modify haplotype frequencies as suggested by Johnson et al. [21]. If we focus on Mexican lionfish (Mexican Caribbean coast and southwest Gulf of Mexico), we find a similar haplotype pattern to other Caribbean localities (S4 Table). Despite the high number of individuals from Mexican sites sequenced in this study (n = 101), we could not identify the rare H03 haplotype reported for the Caribbean region in Grand Cayman, Panama, and Puerto Rico localities at very low frequencies (only one individual at each locality) (S4 Table; [9]). Interestingly, all individuals presenting the H04 haplotype were found on the Caribbean coast of Mexico, while the southwest Gulf of Mexico (Veracruz) only presents H01 and H02 haplotypes. This is surprising when considering the high frequency of H04 haplotype (12%) observed in the northern part of the Gulf of Mexico [21]. Notwithstanding our low sample size, a frequency of 12% should have been sufficient for the observation of some individuals with the H04 haplotype. We argue that the southwest Gulf of Mexico presents a lower frequency for the H04 haplotype, which is more similar to those found in the Caribbean region (around 3%). This hypothesis is supported by the complexity of water circulation presented in this semi-enclosed region of the Gulf of Mexico (Fig 1) with the presence of 1) a cyclonic gyre circulation in the deepest part of the basin of the southern Gulf of Mexico (Campeche Bank) [55], 2) an anticyclonic circulation in the central and north-western part of the Gulf, 3) the presence of eddies in deep waters [56–57], and 4) a semi-decoupling shelf and oceanic waters with coastal inner shelf seasonal circulation off the western Gulf of Mexico dominated by winds [58]. These patterns of circulation dominated by gyres in the western Gulf of Mexico could constrain particles near the coast, maintaining the influence of mesoscale eddies on the outer shelf and resulting in retention areas. These gyre-like oceanic structures situated in front of shelf areas such as Campeche Bank could restrict flow between coastal and oceanic waters but allow a connecting route between coastal areas. Recent investigations into connectivity, based on circulation studies, in the Gulf of Mexico showed a reduced number of pathways between the northern and the southern part of the Gulf [25–26]. To confirm this hypothesis, more individuals from different parts of the Gulf of Mexico need to be sequenced.

The low genetic diversity of lionfish observed in our study using mtDNA was expected due to the founder effect associated with the rapid colonization by an invasive species. Our results were slightly lower but generally consistent with those previously reported [8–9, 36]. However, genetic diversity of lionfish determined by ISSR genetic markers can be considered moderate for the Veracruz locality and high for the remaining sample sites when compared with other ISSR studies of marine species. For example, Casu et al. [54] described a low to medium genetic diversity (*P* from 28 to 40 and *H* from 0.08 to 0.13) for *Diplodus* species (Perciformes, Sparidae), whereas Machkour-M’Rabet et al. [24] revealed high genetic diversity parameters for the queen conch, *Strombus gigas* L. 1758 (Mesogastropoda, Strombidae). It should be noted that the maximum value of expected heterozygosity using dominant genetic markers is 0.5 because only two alleles are produced at each locus [59]. The lowest value observed in Veracruz could be explained by the low sample number (n = 21); but, the site in Cuba, which had the lowest number of individuals (n = 14), exhibited higher genetic diversity. The lower genetic diversity demonstrated by lionfish from the coast of Veracruz (southwest Gulf of Mexico) could reflect a recent founder effect, particularly when considering the very recent invasion [60], as demonstrated for other species [61]. The first lionfish in the southern Gulf of Mexico was observed in late 2009 [62] above the Yucatan shelf on the Alacranes Reef, approximately 700 km (435 mi) from the coast of Veracruz. It has been suggested that due to the circulation pattern in the southern Gulf of Mexico, there is limited connectivity between the Alacranes Reef and other reefs [25]. Based on numerical models, high values of larval retention were observed on the western shelf of the southern Gulf of Mexico [25], reinforcing our findings that the Veracruz site could be relatively isolated from the rest of the system. Higher genetic diversity at the Belize site, could be explained by its particular position with regard to currents that affect the Mesoamerican Reef System. Indeed, the Belize coast is influenced by the Caribbean Current and by the Honduras gyre, this may favor the arrival of individuals from reef areas with different allele frequencies and therefore maintain higher genetic diversity.

Mitochondrial DNA analysis showed no genetic differentiation throughout all lionfish locations considered in this study (*Φ*_*ST*_ and AMOVA results), whereas nuclear ISSR presented a high and significant separation between the southwest Gulf of Mexico and the Caribbean region (*Phi*_*PT*_, AMOVA, and PCoA results). This kind of discrepancy between mitochondrial and nuclear genetic markers in population structure studies has already been demonstrated [63–64], and is primarily due to: 1) a single mtDNA locus have less power to detect population divergence that two or more polymorphic nuclear loci (as ISSR) which are expected more sensitives, 2) higher genetic variability and structure detected by ISSR due to more rapid evolution and a lower degree of influence by natural selection, 3) level of diversity among markers that vary depending species and evolutionary histories, and 4) the limitation in the capacity to detect population divergence may be due to loci with low diversity but also loci with extremely high diversity [63–64 for review]. The genetic break observed between the southwest Gulf of Mexico and the Caribbean region is expected as it has been demonstrated (generally for the Gulf of Mexico) for other marine species in the region such the queen conch (*S*. *gigas*; [24]), the foraging aggregation of the hawksbill turtle (*Eretmochelys imbricata* L. 1766; Testudines, Cheloniideae; [65]), and for the coral *Montastraea annularis* Ellis & Solander 1786 (Scleractinia, Merulinidae; [66]). Recently, Johnson et al. [21], using mtDNA control region, also suggested a restriction in the dispersal of lionfish larvae between the northern Gulf of Mexico and the Caribbean (dashed line -c- on Fig 1 in [21]).

The very recent invasion of the southern area of the Gulf of Mexico, probably remained undetected by mtDNA, while the ISSR genetic markers that are capable of highlighting contemporary events [61, 67–68] confirmed a break between the southwest Gulf of Mexico and the Caribbean region. It is clear that the Yucatan/Loop Current and rings associated with the detachment of the Loop Current that travel westward reaching the Mexican and Texas coasts provides connectivity between the Caribbean and the Gulf of Mexico. Connectivity between these two regions across the Yucatan Shelf is extremely complex [69]; however, the Loop Current appears to clearly connect the Caribbean region (via the Yucatan Current) and the northern Gulf of Mexico as shown recently by Kitchens et al. [27]. These authors used a biophysical dispersal model to backtrack three larval origins found in the northern Gulf of Mexico, and demonstrated that high probability spawning areas would be along the northern edge of the Yucatan Shelf and close to Cozumel Island in the Caribbean Sea. However, considering our results and recent literature [21, 25–27], we suggest that the restricted connectivity between the northern and southern Gulf of Mexico may be attributable to the lack of direct pathways due to a complex circulation pattern (basin gyres such as Campeche Bank Gyre, Campeche Bay Gyre, and Western Gulf Anticyclonic Gyre). Evidently, extensive genetic connectivity studies are essential to confirm and understand the complex connectivity inside the Gulf of Mexico.

An analysis of the Caribbean region using ISSR genetic markers has shown a restricted connectivity between Caribbean locations considered in this study such as the Xcalak and Belize sites, suggesting new breaks in the dispersal capacity of lionfish. Considering the larval period of the lionfish (20–35 days for planktonic dispersal prior to settlement; [70]), this separation could be explained by the spatial restriction of the different oceanographic regimens along the Mesoamerican Barrier Reef System (MBRS) as proposed by Carrillo et al. [31, 71], where the impinging of the Cayman Current separates the southern and northern part of the MBRS. The southern part was characterized by variable and weak currents and the presence of a mesoscale cyclonic gyre, known as the Honduras gyre, which could retain particles and larvae and enhance self-recruitment. The northern MBRS regime, dominated by the strong Yucatan Current, allows rapid dispersal of fish larvae, resulting in a more homogeneous distribution of larvae. This regional scheme for potential connectivity was recently supported by the results obtained from numerical modeling of two species of fish larvae (*Nassau grouper* and *Mutton snapper*) [72].

A previous study [8] suggests no significant correlation between geographical and genetic distances; however, this analysis was on a larger scale and used mtDNA for analysis. Our analysis of the Caribbean region (excluding the Gulf of Mexico), using a highly polymorphic genetic marker (ISSR), suggests an IBD pattern, producing evidence of the effect of geographic distances represented by the significant influence of the Puerto Rico locality. If the Puerto Rico locality is eliminated from the dataset, thus creating a smaller geographic scale, no IBD pattern is demonstrated. The identification of an IBD pattern at a large geographic scale is unsurprising for the lionfish considering that the dispersal capacity of lionfish larvae depends on pelagic larval duration, mortality, and the influence of ocean currents [27, 70, 73].

## Supporting information

S1 Fig Haplotype network for *Pterois volitans*. Each circle represents a haplotype, the size corresponds to haplotype frequency, and the colors correspond to the localities sampled. Brown circles represent other haplotypes present in the Atlantic region, but that were not encountered in this study. Lines connecting haplotypes represent one mutational step and small grey circles denote missing intermediate haplotypes. Localities abbreviation: Puerto Rico (PR), Belize (BZ), Xcalak (XC), Banco Chinchorro (BC), Puerto Morelos (PM), Cuba (CU), Veracruz (VC).
(TIF)

S2 Fig Isolation by distance (IBD) of *Pterois volitans* using ISSR molecular markers in the Caribbean region. (**A**) IBD considering all localities for the Caribbean region, (**B**) IBD at small spatial scale (without Puerto Rico data).
(PDF)

S1 Table GenBank accession numbers for fragment of mtDNA control region genetic marker. (PDF)

S2 Table ISSR data. (XLSX)

S3 Table Analysis of molecular variance (AMOVA) based on nuclear ISSR gene. (A) including all individuals, (B) without individuals from Veracruz locality. Best scenarios are in grey. For details about scenarios tested see Materials and methods or Table 2.
(PDF)

S4 Table Haplotypes proportion (percentage) based on mtDNA control region for the red lionfish, *Pterois volitans*, in the Gulf of Mexico and Caribbean region. Number of individuals (n), haplotype designated (H01-H02-H03-H04).
(PDF)
