# Supplementary figures and images for: Genetic structure of Mexican lionfish populations in the southwest Gulf of Mexico and the Caribbean Sea
Source: PLoS One. 2019 Oct 1;14(10):e0222997. doi: 10.1371/journal.pone.0222997 (PMC6772041; doi:10.1371/journal.pone.0222997)

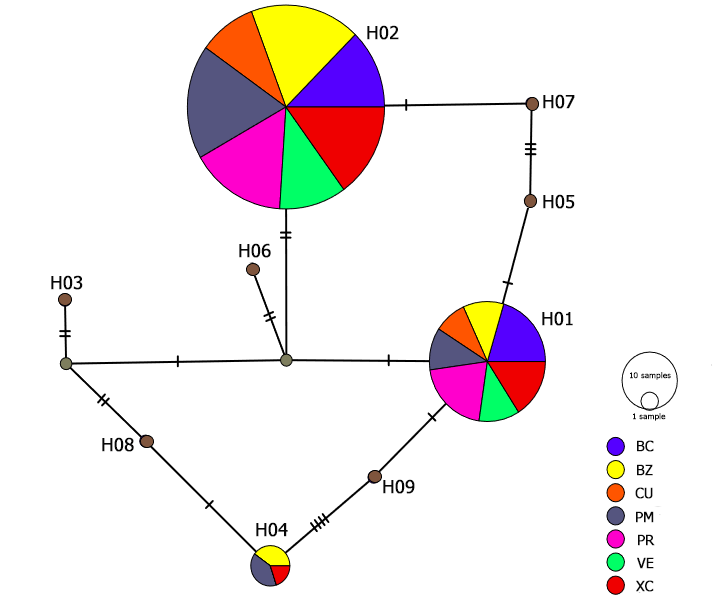

Supplement: S1 Fig — Each circle represents a haplotype, the size corresponds to haplotype frequency, and the colors correspond to the localities sampled. Brown circles represent other haplotypes present in the Atlantic region, but that were not encountered in this study. Lines connecting haplotypes represent one mutational step and small grey circles denote missing intermediate haplotypes. Localities abbreviation: Puerto Rico (PR), Belize (BZ), Xcalak (XC), Banco Chinchorro (BC), Puerto Morelos (PM), Cuba (CU), Veracruz (VC). (TIF) [file pone.0222997.s001.tif]

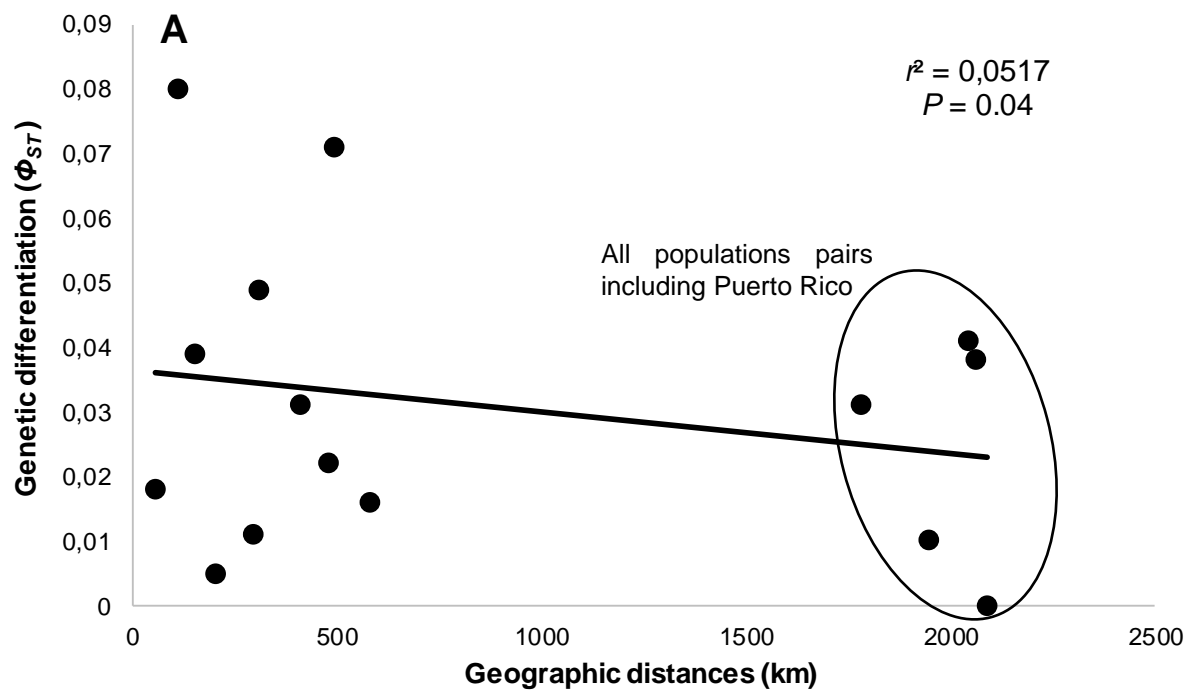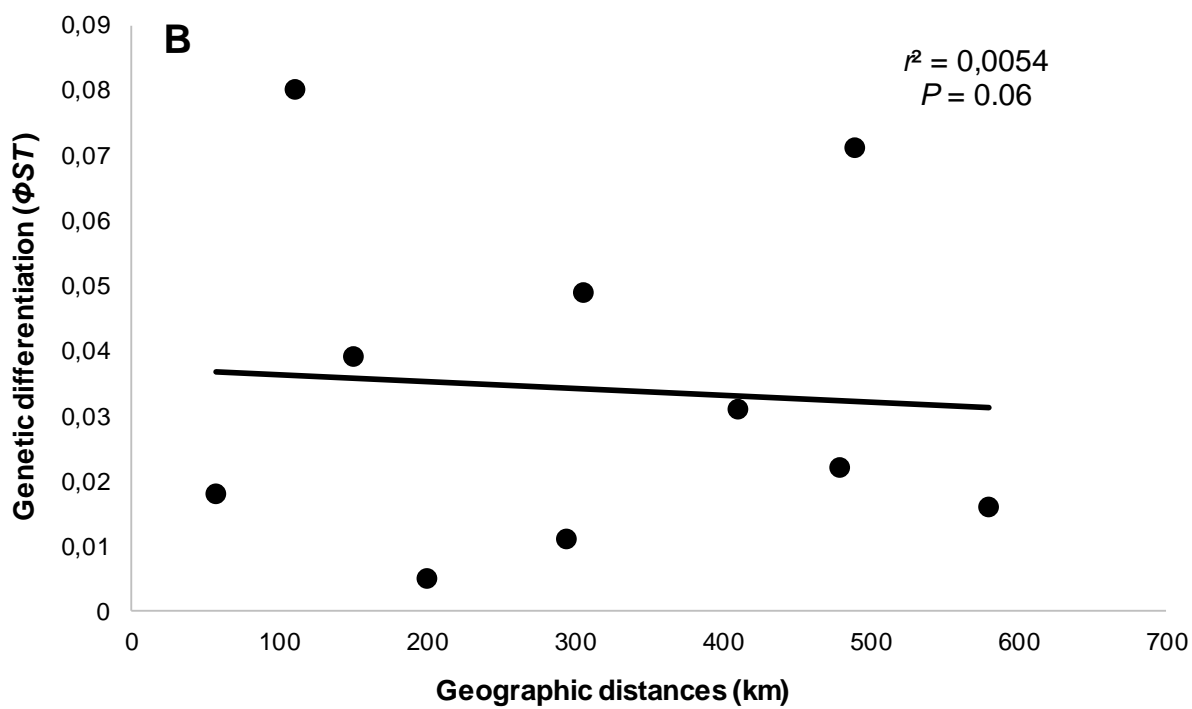

Supplement: S2 Fig — (A) IBD considering all localities for the Caribbean region, (B) IBD at small spatial scale (without Puerto Rico data). (PDF) [file pone.0222997.s002.pdf]
