# Supplementary material for: Genetic structure of Mexican lionfish populations in the southwest Gulf of Mexico and the Caribbean Sea
Source: PLoS One. 2019 Oct 1;14(10):e0222997. doi: 10.1371/journal.pone.0222997 (PMC6772041; doi:10.1371/journal.pone.0222997)
Supplement: S1 Table — (PDF) [file pone.0222997.s003.pdf]

Label ID	Specie	Location	Accession number
BC02	*Pterois volitans*	Coral Negro, RBBCH Q. Roo, Mexico	MN461281
BC03	*Pterois volitans*	Coral Negro, RBBCH Q. Roo, Mexico	MN461282
BC04	*Pterois volitans*	Coral Negro, RBBCH Q. Roo, Mexico	MN461283
BC05	*Pterois volitans*	Coral Negro, RBBCH Q. Roo, Mexico	MN461284
BC08	*Pterois volitans*	Coral Negro, RBBCH Q. Roo, Mexico	MN461285
BC09	*Pterois volitans*	Coral Negro, RBBCH Q. Roo, Mexico	MN461286
BC10	*Pterois volitans*	Acuario II, RBBCH Q. Roo, Mexico	MN461287
BC12	*Pterois volitans*	San Andres, RBBCH Q. Roo, Mexico	MN461288
BC13	*Pterois volitans*	San Andres, RBBCH Q. Roo, Mexico	MN461289
BC18	*Pterois volitans*	Baliza, RBBCH Q. Roo, Mexico	MN461290
BC19	*Pterois volitans*	Baliza, RBBCH Q. Roo, Mexico	MN461291
BC20	*Pterois volitans*	Baliza, RBBCH Q. Roo, Mexico	MN461292
BC21	*Pterois volitans*	Baliza, RBBCH Q. Roo, Mexico	MN461293
BC23	*Pterois volitans*	Baliza, RBBCH Q. Roo, Mexico	MN461294
BC26	*Pterois volitans*	Acuario II, RBBCH Q. Roo, Mexico	MN461295
BC27	*Pterois volitans*	Acuario II, RBBCH Q. Roo, Mexico	MN461296
BC31	*Pterois volitans*	Coral Negro, RBBCH Q. Roo, Mexico	MN461297
BC32	*Pterois volitans*	Coral Negro, RBBCH Q. Roo, Mexico	MN461298
BC33	*Pterois volitans*	Coral Negro, RBBCH Q. Roo, Mexico	MN461299
BC36	*Pterois volitans*	Coral Negro, RBBCH Q. Roo, Mexico	MN461300
BC38	*Pterois volitans*	Coral Negro, RBBCH Q. Roo, Mexico	MN461301
BC39	*Pterois volitans*	Coral Negro, RBBCH Q. Roo, Mexico	MN461302
BC40	*Pterois volitans*	Coral Negro, RBBCH Q. Roo, Mexico	MN461303
BC46	*Pterois volitans*	Coral Negro, RBBCH Q. Roo, Mexico	MN461304
BC52	*Pterois volitans*	González, RBBCH Q. Roo, Mexico	MN461305
BZ03	*Pterois volitans*	Aquarium, Turneffe Atollon MR, Belize	MN461306
BZ05	*Pterois volitans*	Front Dock, Turneffe Atollon MR, Belize	MN461307
BZ07	*Pterois volitans*	Front Dock, Turneffe Atollon MR, Belize	MN461308
BZ10	*Pterois volitans*	Front Dock, Turneffe Atollon MR, Belize	MN461309
BZ14	*Pterois volitans*	Soldier Caye, Turneffe Atollon MR, Belize	MN461310
BZ18	*Pterois volitans*	Soldier Caye, Turneffe Atollon MR, Belize	MN461311
BZ20	*Pterois volitans*	Harry Jones, Turneffe Atollon MR, Belize	MN461312
BZ23	*Pterois volitans*	Harry Jones, Turneffe Atollon MR, Belize	MN461313
BZ24	*Pterois volitans*	Harry Jones, Turneffe Atollon MR, Belize	MN461314
BZ25	*Pterois volitans*	Harry Jones, Turneffe Atollon MR, Belize	MN461315
BZ26	*Pterois volitans*	Harry Jones, Turneffe Atollon MR, Belize	MN461316
BZ28	*Pterois volitans*	Harry Jones, Turneffe Atollon MR, Belize	MN461317

BZ29	*Pterois volitans*	Harry Jones, Turneffe Atollon MR, Belize	MN461318
BZ30	*Pterois volitans*	Front Dock, Turneffe Atollon MR, Belize	MN461319
BZ33	*Pterois volitans*	Calabash, Turneffe Atollon MR, Belize	MN461320
BZ35	*Pterois volitans*	Harry Jones, Turneffe Atollon MR, Belize	MN461321
BZ36	*Pterois volitans*	Harry Jones, Turneffe Atollon MR, Belize	MN461322
BZ38	*Pterois volitans*	Fishing Bogue, Turneffe Atollon MR, Belize	MN461323
BZ40	*Pterois volitans*	Coral gardens, Turneffe Atollon MR, Belize	MN461324
BZ42	*Pterois volitans*	Coral gardens, Turneffe Atollon MR, Belize	MN461325
BZ43	*Pterois volitans*	Coral gardens, Turneffe Atollon MR, Belize	MN461326
BZ44	*Pterois volitans*	Coral gardens, Turneffe Atollon MR, Belize	MN461327
BZ48	*Pterois volitans*	Harry Jones, Turneffe Atollon MR, Belize	MN461328
BZ50	*Pterois volitans*	Harry Jones, Turneffe Atollon MR, Belize	MN461329
BZ51	*Pterois volitans*	Fishing Bogue, Turneffe Atollon MR, Belize	MN461330
BZ52	*Pterois volitans*	Fishing Bogue, Turneffe Atollon MR, Belize	MN461331
BZ53	*Pterois volitans*	Fishing Bogue, Turneffe Atollon MR, Belize	MN461332
BZ54	*Pterois volitans*	Fishing Bogue, Turneffe Atollon MR, Belize	MN461333
BZ55	*Pterois volitans*	Fishing Bogue, Turneffe Atollon MR, Belize	MN461334
BZ56	*Pterois volitans*	Fishing Bogue, Turneffe Atollon MR, Belize	MN461335
CU04	*Pterois volitans*	Laberinto, Guanahacabibes NP, Cuba	MN461336
CU06	*Pterois volitans*	Bajada, Guanahacabibes NP, Cuba	MN461337
CU07	*Pterois volitans*	Alnivonte, Guanahacabibes PN, Cuba	MN461338
CU13	*Pterois volitans*	Bajada, Guanahacabibes PN, Cuba	MN461339
CU18	*Pterois volitans*	Cuevas de Pedro, Guanahacabibes PN, Cuba	MN461340
CU21	*Pterois volitans*	Cuevas de Pedro, Guanahacabibes PN, Cuba	MN461341
CU32	*Pterois volitans*	Laberinto, Guanahacabibes PN, Cuba	MN461342
CU37	*Pterois volitans*	Jardin de las Gorgonias, Guanacahabibes PN, Cuba	MN461343
CU43	*Pterois volitans*	Jardin de las Gorgonias, Guanacahabibes PN, Cuba	MN461344
CU58	*Pterois volitans*	Uvero quemado, Guanacahabibes PN, Cuba	MN461345
CU59	*Pterois volitans*	Alnivonte, Guanahacabibes PN, Cuba	MN461346
CU65	*Pterois volitans*	Laberinto, Guanahacabibes PN, Cuba	MN461347
CU75	*Pterois volitans*	Alnivonte, Guanahacabibes PN, Cuba	MN461348
CU82	*Pterois volitans*	Laberinto, Guanahacabibes PN, Cuba	MN461349
CU92	*Pterois volitans*	Bajada, Guanahacabibes PN, Cuba	MN461350
CU95	*Pterois volitans*	Bajada, Guanahacabibes PN, Cuba	MN461351
PM01	*Pterois volitans*	Punta Brava, B. Juárez, Q. Roo Mexico	MN461352
PM02	*Pterois volitans*	Punta Brava, B. Juárez, Q. Roo Mexico	MN461353
PM04	*Pterois volitans*	Punta Brava, B. Juárez, Q. Roo Mexico	MN461354
PM05	*Pterois volitans*	Punta Brava, B. Juárez, Q. Roo Mexico	MN461355

PM06	*Pterois volitans*	Punta Brava, B. Juárez, Q. Roo Mexico	MN461356
PM07	*Pterois volitans*	Punta Brava, B. Juárez, Q. Roo Mexico	MN461357
PM08	*Pterois volitans*	Punta Brava, B. Juárez, Q. Roo Mexico	MN461358
PM09	*Pterois volitans*	Punta Brava, B. Juárez, Q. Roo Mexico	MN461359
PM10	*Pterois volitans*	Punta Brava, B. Juárez, Q. Roo Mexico	MN461360
PM11	*Pterois volitans*	Punta Brava, B. Juárez, Q. Roo Mexico	MN461361
PM12	*Pterois volitans*	Punta Brava, B. Juárez, Q. Roo Mexico	MN461362
PM13	*Pterois volitans*	Punta Brava, B. Juárez, Q. Roo Mexico	MN461363
PM14	*Pterois volitans*	Punta Brava, B. Juárez, Q. Roo Mexico	MN461364
PM17	*Pterois volitans*	Picudas, B. Juárez, Q. Roo, Mexico	MN461365
PM18	*Pterois volitans*	Picudas, B. Juárez, Q. Roo, Mexico	MN461366
PM19	*Pterois volitans*	Picudas, B. Juárez, Q. Roo, Mexico	MN461367
PM20	*Pterois volitans*	Picudas, B. Juárez, Q. Roo, Mexico	MN461368
PM22	*Pterois volitans*	Picudas, B. Juárez, Q. Roo, Mexico	MN461369
PM24	*Pterois volitans*	Punta Nizuc, Cancun, Q. Roo, Mexico	MN461370
PM25	*Pterois volitans*	Punta Nizuc, Cancun, Q. Roo, Mexico	MN461371
PM26	*Pterois volitans*	Punta Nizuc, Cancun, Q. Roo, Mexico	MN461372
PM27	*Pterois volitans*	Punta Nizuc, Cancun, Q. Roo, Mexico	MN461373
PM28	*Pterois volitans*	Punta Nizuc, Cancun, Q. Roo, Mexico	MN461374
PM30	*Pterois volitans*	Punta Nizuc, Cancun, Q. Roo, Mexico	MN461375
PM31	*Pterois volitans*	Punta Nizuc, Cancun, Q. Roo, Mexico	MN461376
PM36	*Pterois volitans*	Punta Nizuc, Cancun, Q. Roo, Mexico	MN461377
PM37	*Pterois volitans*	Punta Nizuc, Cancun, Q. Roo, Mexico	MN461378
PM38	*Pterois volitans*	Punta Nizuc, Cancun, Q. Roo, Mexico	MN461379
PM40	*Pterois volitans*	Punta Nizuc, Cancun, Q. Roo, Mexico	MN461380
PM43	*Pterois volitans*	Punta Nizuc, Cancun, Q. Roo, Mexico	MN461381
PR01	*Pterois volitans*	La Parguera, Puerto Rico	MN461382
PR02	*Pterois volitans*	La Parguera, Puerto Rico	MN461383
PR03	*Pterois volitans*	La Parguera, Puerto Rico	MN461384
PR04	*Pterois volitans*	La Parguera, Puerto Rico	MN461385
PR05	*Pterois volitans*	La Parguera, Puerto Rico	MN461386
PR06	*Pterois volitans*	La Parguera, Puerto Rico	MN461387
PR07	*Pterois volitans*	La Parguera, Puerto Rico	MN461388
PR08	*Pterois volitans*	La Parguera, Puerto Rico	MN461389
PR09	*Pterois volitans*	La Parguera, Puerto Rico	MN461390
PR10	*Pterois volitans*	La Parguera, Puerto Rico	MN461391
PR11	*Pterois volitans*	La Parguera, Puerto Rico	MN461392
PR12	*Pterois volitans*	La Parguera, Puerto Rico	MN461393

PR15	*Pterois volitans*	La Parguera, Puerto Rico	MN461394
PR16	*Pterois volitans*	La Parguera, Puerto Rico	MN461395
PR17	*Pterois volitans*	La Parguera, Puerto Rico	MN461396
PR20	*Pterois volitans*	La Parguera, Puerto Rico	MN461397
PR22	*Pterois volitans*	La Parguera, Puerto Rico	MN461398
PR24	*Pterois volitans*	La Parguera, Puerto Rico	MN461399
PR25	*Pterois volitans*	La Parguera, Puerto Rico	MN461400
PR29	*Pterois volitans*	La Parguera, Puerto Rico	MN461401
PR30	*Pterois volitans*	La Parguera, Puerto Rico	MN461402
PR31	*Pterois volitans*	La Parguera, Puerto Rico	MN461403
PR32	*Pterois volitans*	La Parguera, Puerto Rico	MN461404
PR33	*Pterois volitans*	La Parguera, Puerto Rico	MN461405
PR34	*Pterois volitans*	La Parguera, Puerto Rico	MN461406
PR36	*Pterois volitans*	La Parguera, Puerto Rico	MN461407
PR38	*Pterois volitans*	La Parguera, Puerto Rico	MN461408
PR39	*Pterois volitans*	La Parguera, Puerto Rico	MN461409
PR40	*Pterois volitans*	La Parguera, Puerto Rico	MN461410
VE01	*Pterois volitans*	Gallegilla PNSAV, Veracruz, Mexico	MN461411
VE02	*Pterois volitans*	Arrecife de Pajaros, PNSAV, Veracruz, Mexico	MN461412
VE03	*Pterois volitans*	La Palma, PNSAV, Veracruz, Mexico	MN461413
VE04	*Pterois volitans*	Arrecife de Pajaros, PNSAV, Veracruz, Mexico	MN461414
VE06	*Pterois volitans*	Santiaguillo, PNSAV, Veracruz, Mexico	MN461415
VE07	*Pterois volitans*	La Palma, PNSAV, Veracruz, Mexico	MN461416
VE08	*Pterois volitans*	Santiaguillo, PNSAV, Veracruz, Mexico	MN461417
VE09	*Pterois volitans*	Blanquilla, PNSAV, Veracruz, Mexico	MN461418
VE10	*Pterois volitans*	Isla Sacrificios, PNSAV, Veracruz, Mexico	MN461419
VE11	*Pterois volitans*	Gallega PNSAV, Veracruz, Mexico	MN461420
VE13	*Pterois volitans*	Gallega PNSAV, Veracruz, Mexico	MN461421
VE14	*Pterois volitans*	Anegada de afuera PNSAV, Veracruz, Mexico	MN461422
VE15	*Pterois volitans*	Arrecife de Pajaros, PNSAV, Veracruz, Mexico	MN461423
VE16	*Pterois volitans*	Santiaguillo, PNSAV, Veracruz, Mexico	MN461424
VE17	*Pterois volitans*	Santiaguillo, PNSAV, Veracruz, Mexico	MN461425
VE18	*Pterois volitans*	Mersey, PNSAV, Veracruz, Mexico	MN461426
VE19	*Pterois volitans*	Sacrificios, PNSAV, Veracruz, Mexico	MN461427
VE20	*Pterois volitans*	Sargazo, PNSAV, Veracruz, Mexico	MN461428
VE21	*Pterois volitans*	Arrecife de Pajaros, PNSAV, Veracruz, Mexico	MN461429
XC02	*Pterois volitans*	Arrecifes de Xcalak, PN, Q. Roo Mexico	MN461430
XC03	*Pterois volitans*	Arrecifes de Xcalak, PN, Q. Roo Mexico	MN461431

XC05	*Pterois volitans*	Arrecifes de Xcalak, PN, Q. Roo Mexico	MN461432
XC06	*Pterois volitans*	Arrecifes de Xcalak, PN, Q. Roo Mexico	MN461433
XC07	*Pterois volitans*	Arrecifes de Xcalak, PN, Q. Roo Mexico	MN461434
XC16	*Pterois volitans*	Arrecifes de Xcalak, PN, Q. Roo Mexico	MN461435
XC53	*Pterois volitans*	Arrecifes de Xcalak, PN, Q. Roo Mexico	MN461436
XC56	*Pterois volitans*	Arrecifes de Xcalak, PN, Q. Roo Mexico	MN461437
XC57	*Pterois volitans*	Arrecifes de Xcalak, PN, Q. Roo Mexico	MN461438
XC58	*Pterois volitans*	Arrecifes de Xcalak, PN, Q. Roo Mexico	MN461439
XC61	*Pterois volitans*	Arrecifes de Xcalak, PN, Q. Roo Mexico	MN461440
XC65	*Pterois volitans*	Arrecifes de Xcalak, PN, Q. Roo Mexico	MN461441
XC66	*Pterois volitans*	Arrecifes de Xcalak, PN, Q. Roo Mexico	MN461442
XC67	*Pterois volitans*	Arrecifes de Xcalak, PN, Q. Roo Mexico	MN461443
XC71	*Pterois volitans*	Arrecifes de Xcalak, PN, Q. Roo Mexico	MN461444
XC74	*Pterois volitans*	Arrecifes de Xcalak, PN, Q. Roo Mexico	MN461445
XC75	*Pterois volitans*	Arrecifes de Xcalak, PN, Q. Roo Mexico	MN461446
XC76	*Pterois volitans*	Arrecifes de Xcalak, PN, Q. Roo Mexico	MN461447
XC77	*Pterois volitans*	Arrecifes de Xcalak, PN, Q. Roo Mexico	MN461448
XC78	*Pterois volitans*	Arrecifes de Xcalak, PN, Q. Roo Mexico	MN461449
XC79	*Pterois volitans*	Arrecifes de Xcalak, PN, Q. Roo Mexico	MN461450
XC80	*Pterois volitans*	Arrecifes de Xcalak, PN, Q. Roo Mexico	MN461451
XC81	*Pterois volitans*	Arrecifes de Xcalak, PN, Q. Roo Mexico	MN461452
XC82	*Pterois volitans*	Arrecifes de Xcalak, PN, Q. Roo Mexico	MN461453
XC85	*Pterois volitans*	Arrecifes de Xcalak, PN, Q. Roo Mexico	MN461454
XC88	*Pterois volitans*	Arrecifes de Xcalak, PN, Q. Roo Mexico	MN461455
XC89	*Pterois volitans*	Arrecifes de Xcalak, PN, Q. Roo Mexico	MN461456
