# Supplementary material for: Genetic structure of Mexican lionfish populations in the southwest Gulf of Mexico and the Caribbean Sea
Source: PLoS One. 2019 Oct 1;14(10):e0222997. doi: 10.1371/journal.pone.0222997 (PMC6772041; doi:10.1371/journal.pone.0222997)
Supplement: S3 Table — (A) including all individuals, (B) without individuals from Veracruz locality. Best scenarios are in grey. For details about scenarios tested see Materials and methods or Table 2. (PDF) [file pone.0222997.s005.pdf]

Source of variation	df	SS	MS	Est. Variance	% variance	*P*
**A**						
Scenario 1						
Among pops	6	269,517	44,919	1,109	8%	0.0001
Within pops	203	2463,093	12,133	12,133	92%	
Total	209	2732,610		13,242	100%	
Scenario 2						
Among regions	1	143,795	143,795	3,236	20%	0.0001
Among pops	5	125,722	25,144	0,419	3%	0.0001
Within pops	203	2463,093	12,133	12,133	77%	0.0001
Total	209	2732,610		15,789	100%	
Scenario 3						
Among regions	2	162,646	81,323	0,921	7%	0.0001
Among pops	4	106,871	26,718	0,479	4%	0.0001
Within pops	203	2463,093	12,133	12,133	90%	0.0001
Total	209	2732,610		13,533	100%	
Scenario 4						
Among regions	2	158,076	79,038	1,139	8%	0.0001
Among pops	4	111,441	27,860	0,489	4%	0.0001
Within pops	203	2463,093	12,133	12,133	88%	0.0001
Total	209	2732,610		13,761	100%	
**B**						
All localities together						
Among pops	5	125,722	25,144	0,411	3,21%	0.0001
Within pops	183	2269,283	12,400	12,400	96,79%	
Total	188	2395,005		12,811	100,00%	
Coasts (XC/PM/BZ) vs Caribbean (CU/PR/BC)						

Among regions	1	18,851	18,851	0,000	0,00%	1.000
Among pops	4	106,871	26,718	0,470	3,65%	0.0001
Within pops	183	2269,283	12,400	12,400	96,35%	0.0001
Total	188	2395,005		12,871	100,00%	
Mx/BZ Caribbean vs Caribbean (CU/PR)						
Among regions	1	14,281	14,281	0,000	0,00%	1.000
Among pops	4	111,441	27,860	0,480	3,73%	0.0001
Within pops	183	2269,283	12,400	12,400	96,27%	0.0001
Total	188	2395,005		12,881	100,00%	
CU/PR/BC/PM vs XC vs BZ						
Among Regions	2	78,582	39,291	0,437	3,37%	0,0001
Among Pops	3	47,140	15,713	0,119	0,92%	0,0353
Within Pops	183	2269,283	12,400	12,400	95,71%	0,0001
Total	188	2395,005		12,956	100,00%	
