# Supplementary material for: Genetic structure of Mexican lionfish populations in the southwest Gulf of Mexico and the Caribbean Sea
Source: PLoS One. 2019 Oct 1;14(10):e0222997. doi: 10.1371/journal.pone.0222997 (PMC6772041; doi:10.1371/journal.pone.0222997)
Supplement: S4 Table — Number of individuals (n), haplotype designated (H01-H02-H03-H04). (PDF) [file pone.0222997.s006.pdf]

	Country	n	H01(%)	H02(%)	H03(%)	H04(%)	Reference
**Gulf of Mexico**	**US (north)**	188	27.7	60.1	0.0	12.2	Johnson et al. 2016
	**México (south)**	21	26.3	73.7	0.0	0.0	This study
**Caribbean**	**Mexico**	82	25.6	70.7	0.0	3.7	This study
	**Belize**	30	16.7	76.7	0.0	6.6	This study
	**Belize**	59	28.8	66.1	0.0	5.1	Butterfield et al. 2015
	**Cuba**	16	25.0	75.0	0.0	0.0	This study
	**Cuba**	24	20.8	75.0	0.0	4.2	Butterfield et al. 2015
	**Gran Caiman**	79	26.6	70.8	1.3	1.3	Betancur-R et al. 2011
	**Jamaica**	37	18.9	75.7	0.0	5.4	Butterfield et al. 2015
	**Honduras**	15	33.3	53.3	0.0	13.3	Butterfield et al. 2015
	**Santa Andrés Islands**	47	38.3	55.3	0.0	6.4	Betancur-R et al. 2011
	**Panama**	5	0.0	80.0	20.0	0.0	Butterfield et al. 2015
	**Santa Marta**	166	25.3	63.8	0.0	10.8	Betancur-R et al. 2011
	**Bonaire**	21	19.0	80.9	0.0	0.0	Butterfield et al. 2015
	**Puerto Rico**	29	31.0	69.0	0.0	0.0	This study
	**Puerto Rico**	138	28.3	68.8	0.7	2.2	Butterfield et al. 2015<br>Toledo-Hernandéz et al. 2014
	**US Virgin Islands**	10	10.0	80.0	0.0	10.0	Butterfield et al. 2015
